# Supplementary material for: Habitat Suitability and Determinants for Anatidae in Multi-Watershed Composite Wetlands in Anhui, China
Source: Animals (Basel). 2024 Mar 26;14(7):1010. doi: 10.3390/ani14071010 (PMC11010902; doi:10.3390/ani14071010)
Supplement: Supplementary file 1 [file animals-14-01010-s001.zip › Figure S1.pdf]

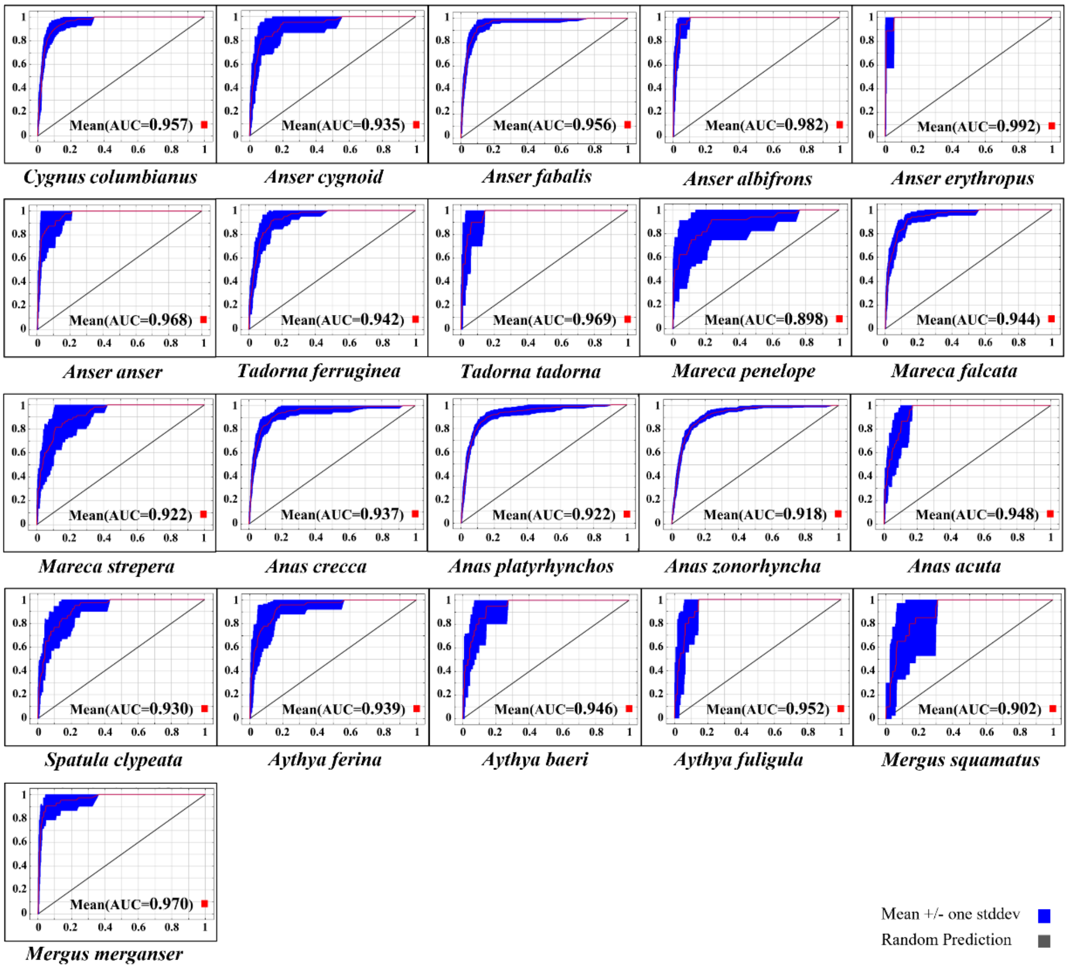

Figure S1. ROC curve and AUC values for 21 Anatidae species from MaxEnt models.

X: Fractional Predicted Area, Y: Sensitivity
