# Supplementary material for: Habitat Suitability and Determinants for Anatidae in Multi-Watershed Composite Wetlands in Anhui, China
Source: Animals (Basel). 2024 Mar 26;14(7):1010. doi: 10.3390/ani14071010 (PMC11010902; doi:10.3390/ani14071010)
Supplement: Supplementary file 1 [file animals-14-01010-s001.zip › Table S1.pdf]

Table S1. Environmental variables for MaxEnt and GLMM models.

Category	Code	Description	Source
Climate variables	bio1	Annual mean temperature	WorldClim
	bio2	Mean diurnal rang	
	bio3	Isothermality	
	bio4	Temperature seasonality	
	bio5	Max temperature of warmest month	
	bio6	Min temperature of coldest month	
	bio7	Temperature annual range	
	bio8	Mean temperature of wettest quarter	
	bio9	Mean temperature of driest quarter	
	bio10	Mean temperature of warmest quarter	
	bio11	Mean temperature of coldest quarter	
	bio12	Annual precipitation	
	bio13	Precipitation of wettest month	
	bio14	Precipitation of driest month	
	bio15	Precipitation seasonality	
	bio16	Precipitation of wettest quarter	
	bio17	Precipitation of driest quarter	
	bio18	Precipitation of warmest quarter	
	bio19	Precipitation of coldest quarter	
Land use	LU	Land use/cover	Resource and Environment Science and Data Center
	DF	Distribution of farmland	Ministry of Education Ecological Field Station for East Asian Migratory Birds
Topography	DEM	Altitude	Geospatial Data Cloud
Human disturbance	RD	Distance to road	OpenStreetMap
	POP	Distribution of the population	Resource and Environment Science and Data Center
	CD	Distance to countryside	Resource and Environment Science and Data Center
	HF	Human footprint	China Agricultural University
