# Supplementary material for: Habitat Suitability and Determinants for Anatidae in Multi-Watershed Composite Wetlands in Anhui, China
Source: Animals (Basel). 2024 Mar 26;14(7):1010. doi: 10.3390/ani14071010 (PMC11010902; doi:10.3390/ani14071010)
Supplement: Supplementary file 1 [file animals-14-01010-s001.zip › Table S2.pdf]

Table S2. Variables retained from the final modelling for each species.

Species	Reserved variables
Cygnus columbianus	bio1, bio5, bio8, bio13, bio14, bio15, bio16, bio17, bio18, bio19, LU, HF, DF, DEM
Anser cygnoid	bio1, bio5, bio13, bio14, bio15, bio16, bio17, bio18, bio19, LU, POP, DF, CD
Anser fabalis	bio1, bio5, bio8, bio13, bio14, bio15, bio16, bio17, bio18, bio19, LU, DF, DEM
Anser albifrons	bio1, bio5, bio10, bio13, bio14, bio15, bio16, bio17, bio18, bio19, DEM, HF, DF, CD
Anser erythropus	bio2, bio5, bio13, bio14, bio15, bio16, bio17, bio18, bio19, LU, POP, DF, RD
Anser anser	bio1, bio5, bio8, bio13, bio14, bio15, bio16, bio17, bio18, bio19, POP, DF, RD, CD
Tadorna ferruginea	bio1, bio5, bio13, bio14, bio15, bio16, bio17, bio18, bio19, LU, HF, DEM
Tadorna tadorna	bio1, bio5, bio13, bio14, bio15, bio16, bio17, bio18, bio19, LU, DEM, RD
Mareca penelope	bio1, bio5, bio13, bio14, bio15, bio16, bio17, bio18, bio19, LU, HF, RD
Mareca falcata	bio1, bio5, bio13, bio14, bio15, bio16, bio17, bio18, bio19, LU, POP, DF
Mareca strepera	bio1, bio5, bio13, bio14, bio15, bio16, bio17, bio18, bio19, LU, DEM, CD
Anas crecca	bio1, bio5, bio13, bio14, bio15, bio16, bio17, bio18, bio19, LU, HF, DF, DEM
Anas platyrhynchos	bio5, bio13, bio14, bio15, bio16, bio17, bio18, bio19, LU, DEM, POP, DF
Anas zonorhycha	bio5, bio13, bio14, bio15, bio16, bio17, bio18, bio19, LU, HF, POP, DF
Anas acuta	bio1, bio5, bio13, bio14, bio15, bio16, bio17, bio18, bio19, LU, DEM, RD
Spatula clypeata	bio1, bio5, bio13, bio14, bio15, bio16, bio17, bio18, bio19, RD, HF, CD, DEM
Aythya ferina	bio1, bio5, bio13, bio14, bio15, bio16, bio17, bio18, bio19, LU, DEM, RD
Aythya baeri	bio1, bio5, bio8, bio13, bio14, bio15, bio16, bio17, bio18, bio19, LU, HF, DF
Aythya fuligula	bio1, bio5, bio13, bio14, bio15, bio16, bio17, bio18, bio19, LU, POP, DF, DEM
Mergus squamatus	bio1, bio5, bio8, bio13, bio14, bio15, bio16, bio17, bio18, bio19, LU, DF, RD
Mergus merganser	bio1, bio5, bio10, bio13, bio14, bio15, bio16, bio17, bio18, bio19, DEM, HF, DF

bio1 – 19: 19 bioclimatic variables, DEM: digital elevation model, HF: human footprint, DF: distribution of farmland, POP: population data, CD: distance to countryside, RD: distance to road.
