# Supplementary figures and images for: Habitat Suitability and Determinants for Anatidae in Multi-Watershed Composite Wetlands in Anhui, China
Source: Animals (Basel). 2024 Mar 26;14(7):1010. doi: 10.3390/ani14071010 (PMC11010902; doi:10.3390/ani14071010)

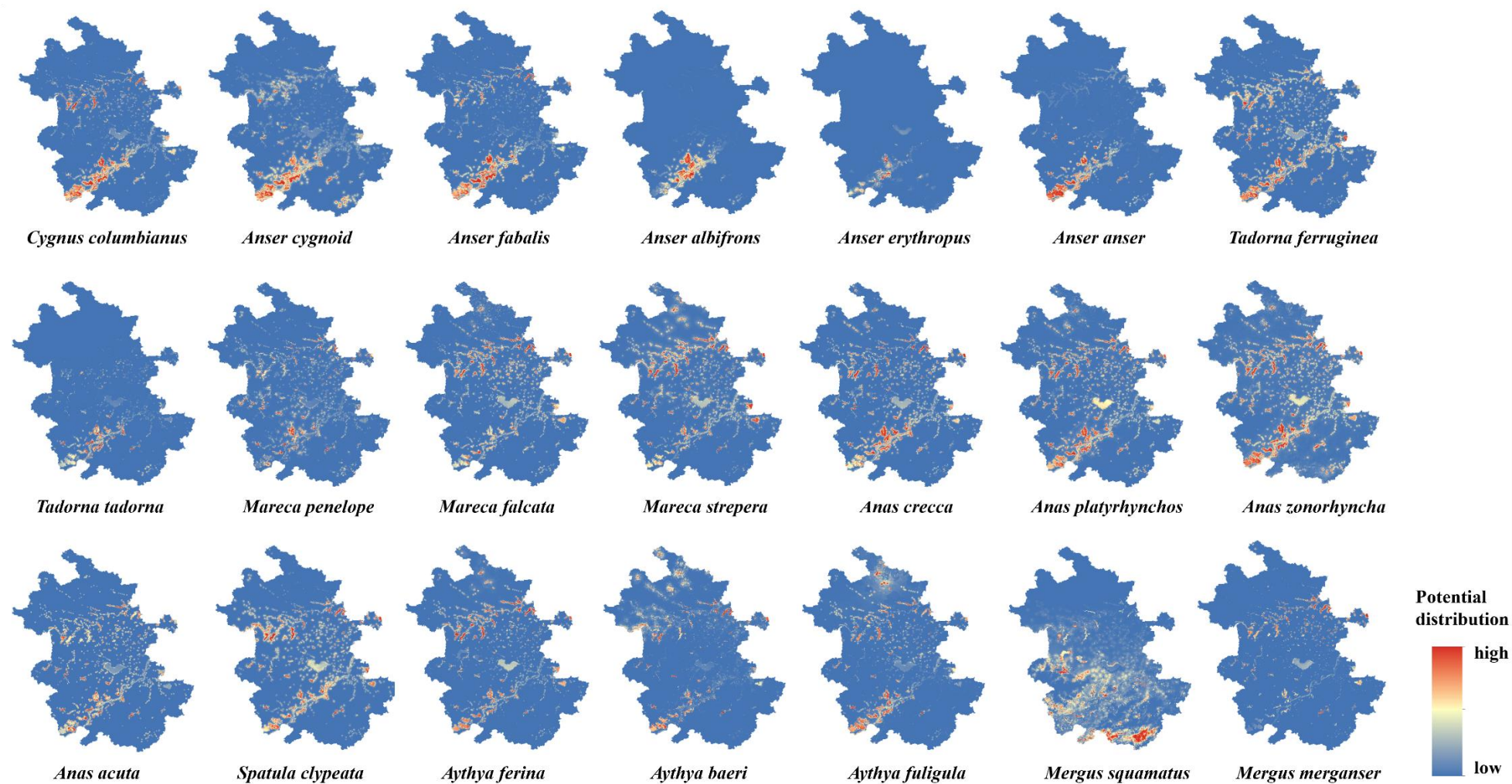

Figure S2. Potential distribution for 21 Anatidae species from Maxent models.

Supplement: Supplementary file 1 [file animals-14-01010-s001.zip › Figure S2.pdf]
